# Familial occurrence of autoimmune liver disease with overlapping features of primary biliary cholangitis and autoimmune hepatitis in a mother and her daughter

**DOI:** 10.1007/s12328-016-0676-1

**Published:** 2016-08-08

**Authors:** Kaoru Omori, Kanako Yoshida, Masaki Yokota, Tsutomu Daa, Masahiro Kan

**Affiliations:** 1 Department of Gastroenterology and Hepatology, Sato Daiichi Hospital, 77-1 Hokyoji, Usa, Oita 879-0454 Japan; 2 Department of Gastroenterology, Nakatsu Municipal Hospital, 173 Shimo-Ikenaga, Nakatsu, Oita 871-8511 Japan; 3 Department of Pathology, Graduate School of Medicine, Faculty of Medicine, Oita University, 1-1 Idaigaoka, Hasama-machi, Yufu, Oita 879-5593 Japan

**Keywords:** Primary biliary cholangitis, Autoimmune hepatitis, Overlap syndrome, Familial

## Abstract

We encountered two patients with overlapping features of primary biliary cholangitis and autoimmune hepatitis within the same family. A 68-year-old woman presented at our hospital from a previous medical institution because of the diagnosis of primary biliary cholangitis. Her 49-year-old daughter was admitted with liver dysfunction 4 years later. When compared, these two related patients were found to have overlapping features of primary biliary cholangitis and autoimmune hepatitis. Their human leukocyte antigen haplotype was DRB1*04:05/DRB1*15:02. The clinical and biochemical findings of these two patients immediately improved following treatment with a combination of prednisolone and ursodeoxycholic acid, in accordance with the Japanese guidelines. It is extremely important to identify such pathological conditions as quickly as possible, particularly with the appearance of severe liver dysfunction due to liver cirrhosis, as observed in our case. The Japanese guidelines are considered to be a realistic and useful clinical policy for the swift and efficient treatment of patients with overlapping features of primary biliary cholangitis and autoimmune hepatitis. We suggest that our two patients presented with a genetic predisposition to autoimmune liver disease with overlapping features of primary biliary cholangitis and autoimmune hepatitis within the same family.

## Introduction

Primary biliary cholangitis (PBC) and autoimmune hepatitis (AIH) are both considered to be autoimmune diseases of the liver. Although they share the autoimmune aspect, their pathogenesis, clinical features, disease course, and response to therapy are quite distinct. PBC is a slowly progressive disease primarily of the intrahepatic bile ducts, with a strong preponderance of females and a median age of 50 years at diagnosis. However, familial PBC is rare (~1–6 % of PBC cases) and an earlier study suggested that human leukocyte antigen (HLA)-DR8 represents a risk factor for developing PBC [1]. Therapy with ursodeoxycholic acid (UDCA) appears to slow the progression of this disease. On the other hand, AIH is a chronic disorder characterized by continuing hepatocellular necrosis and inflammation, usually with fibrosis, which can evolve into liver cirrhosis and failure. AIH commonly affects young women, who usually exhibit characteristic autoantibodies and a characteristic immunogenetic background involving HLA-B8, DR3, or DR4 [2]. Familial cases of AIH are reported to occur in only 1 % of AIH cases [3]. Moreover, response to immunosuppressive therapy is usually prompt in this disease, resulting in a good long-term prognosis [4].

Patients with overlapping features of PBC and AIH have been recognized for some time. Although the prevalence of autoimmune liver disease with overlapping features of PBC and AIH has been reported to vary considerably (2.1–19 %) [5], there are few reports of cases occurring within the same family. Here we report overlapping features of PBC and AIH in the mother and daughter of the same family.

## Case report

**Fig. 1:**
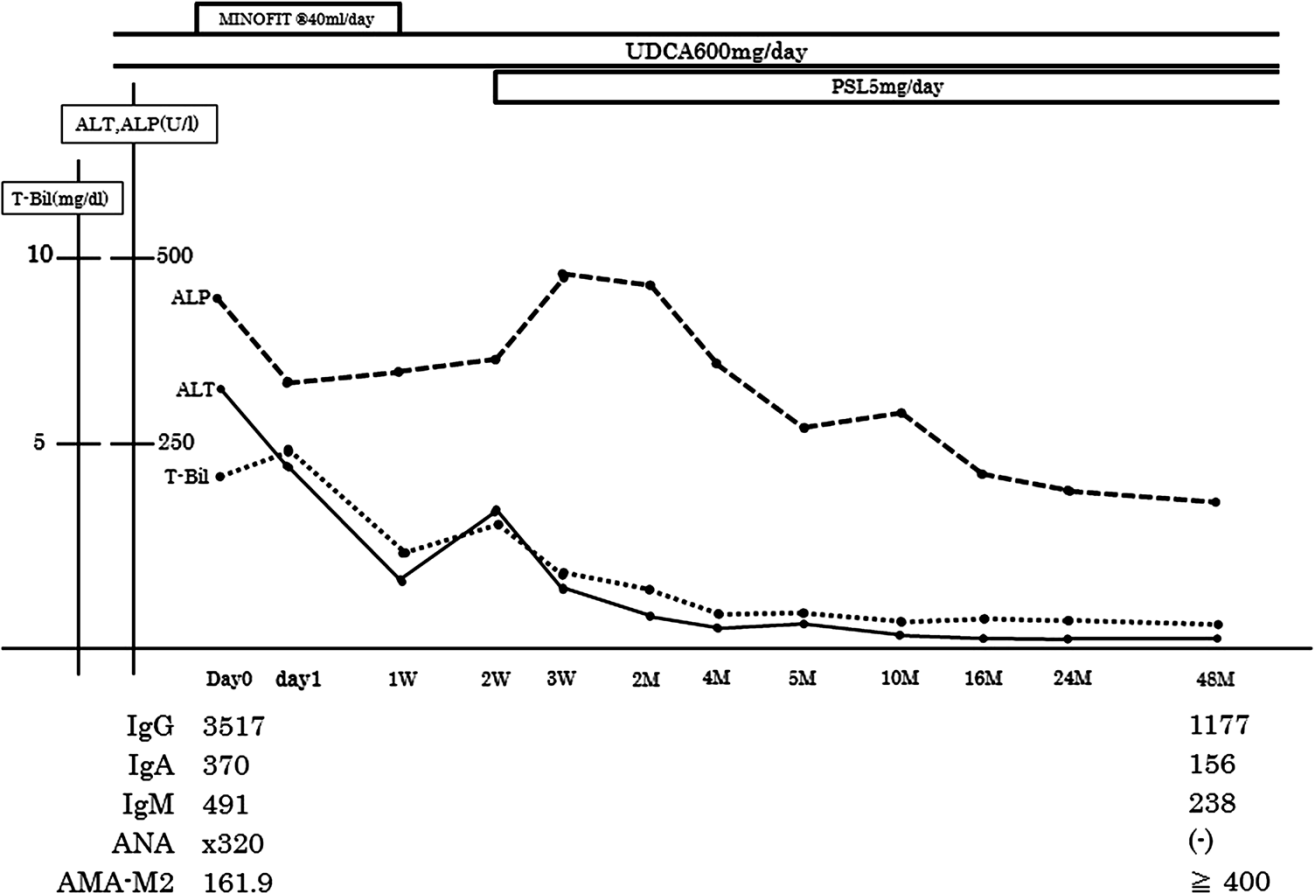
Clinical course of case 1 (mother). Following diagnosis with an autoimmune liver disease with overlapping features of primary biliary cholangitis and autoimmune hepatitis, the patient was treated with a combination of 600 mg/day ursodeoxycholic acid (*UDCA*) and 5 mg/day prednisolone (*PSL*) in accordance with the Japanese guidelines, resulting in the gradual improvement of both clinical and biochemical findings. Key: *ANA* antinuclear antibody, *AMA-M2* anti-mitochondrial antibody-M2

Case 1: a 68-year-old woman was admitted to a previous medical institution because of liver dysfunction. She was diagnosed with PBC by the following biochemical and serological findings: aspartate aminotransferase (AST) 212 U/L, alanine aminotransferase (ALT) 203 U/L, total bilirubin (T-Bil) 0.9 mg/dL, alkaline phosphatase (ALP) 491 U/L, γ-glutamyl transpeptidase (γ-GTP) 212 U/L, immunoglobulin (Ig) M 534 mg/dL, and anti-mitochondrial antibody (AMA) titer 1:80. Ultrasonography (US) and computed tomography (CT) showed morphological changes indicative of liver cirrhosis. Although the patient was immediately treated with 600 mg UDCA, the response to this treatment was not sufficient. She was referred by another medical institution to our hospital for further, more detailed examinations and was subsequently admitted to our hospital due to further liver dysfunction and progressive symptoms over the previous 4 weeks, including general fatigue, appetite loss, and muscle cramp in her legs. Her initial laboratory tests revealed liver dysfunction with an antinuclear antibody (ANA) titer of 1:320 with a homogeneous pattern, and elevation of IgG to 3,517 mg/dL (Table 1). Serological examinations for hepatitis A virus (HAV), hepatitis B virus (HBV), and hepatitis C virus (HCV) were all negative (Table 1). There was no history of symptoms suggestive of viral infection. International AIH score on the basis of the simplified International Autoimmune Hepatitis Group (IAIHG) scoring system and the revised AIH scoring system was 6 points and 13 points, respectively, implying a probable AIH [6, 7]. The HLA-DRB1 haplotype was DRB1*04:05/DRB1*15:02. Although the patient had ingested some health foods, liver dysfunction had not improved when these foods were stopped. Drug-induced lymphocyte stimulation tests for such food types were negative. Thus, the patient was eventually diagnosed on the basis of both the Paris criteria and Japanese guidelines with an autoimmune liver disease with overlapping features of PBC and AIH [4, 8]. We immediately administered 5 mg/day prednisolone (PSL) and 600 mg/day of UDCA [9]. The PSL dose given was smaller than that indicated by the Japanese guidelines because of the complication of liver cirrhosis and the consequent increased risk of opportunistic infections, osteoporosis, and abnormalities in glucose metabolism. This course of treatment resulted in improvements to clinical and biochemical findings within 1 month. Since then, liver function has remained within the normal range, resulting in a constant liver cirrhotic condition of Child–Pugh class A (Fig. 1). Furthermore, the current level of bone mineral density (BMD) is 0.396 g/cm^2^ greater than the normal range (0.261–0.333 g/cm^2^), although we continue to administer PSL at a dose of 5 mg/day.

**Table 1:** Laboratory data at the first medical examination and comparison of environmental and genetic factors between all family members

	Case 1 (mother)	Case 2 (daughter 1)	Daughter 2
Age (years)	68	49	48
Biochemistry			
TP (6.5–8.3) (g/dL)	7.8	8.4	7.3
Alb (3.8–5.3) (g/dL)	2.9	3.9	4.4
T.Bil (0.2–1.2) (mg/dL)	4.25	4.79	0.56
AST (5–40) (IU/L)	328	1203	13
ALT (3–35) (IU/L)	312	1425	10
γ-GTP (10–60) (IU/L)	60	208	20
ALP (100–340) (IU/L)	418	900	149
Hematology			
WBC (4000–8000) (/μL)	3400	5100	6400
RBC (350–480) (×10^4^/μL)	316	446	436
Hb (11.5–16.0) (g/dL)	10.8	14.7	13.8
Plt (12.0–40.0) (×10^4^/uL)	11.8	21.1	26.6
PT % (%)	41.6	79.8	94.0
APTT (26–38) (s)	41.0	35.1	27.7
Serology			
IgG (820–1740) (mg/dL)	3517	2261	1249
IgA (90–400) (mg/dL)	370	386	121
IgM (52–270) (mg/dL)	491	203	173
ANA (0–79)	×320	×80	<40
AMA (0–19)	(×80)	×40	Not tested
AMA-M2 (0.0–6.9)	161.9	55.8	75.7
ASMA (0–19)	<20	<20	Not tested
Anti-LKM1 (0–16.9)	<5.0	5.2	Not tested
Viral marker			
IgM anti-HA	–	–	Not tested
HBs-Ag	–	–	–
HCV-Ab	–	–	–
IgM anti-HBc (0–0.9)	(−)	–	Not tested
IgG anti-HBc (0–0.99)	(−)	–	Not tested
HCV–RNA (PCR)	(−)	–	Not tested
HLA-DRB1 haplotype	DRB1*04:05, DRB1*15:02	DRB1*04:05, DRB1*15:02	DRB1*04:05, DRB1*01:01
Smoking status/alcohol consumption	Non-smoker/nil	Non-smoker/nil	Non-smoker/nil
Occupation	Housewife 18 years–present	Nurse 20 years–present	Nurse 20 years–present
Period of time living with mother		Birth–20 years	Birth–20 years
Previous medical history	Unremarkable	Unremarkable	Unremarkable
Medication use	Nil	Nil	Nil
Obstetric history	2 deliveries	3 deliveries	2 deliveries
Serological evidence of past infection			
Epstein–barr virus	+	+	Not tested
Cytomegalovirus	+	+	Not tested

(−) Serological findings of a previous medical institution, *ANA* antinuclear antibody, *AMA* anti-mitochondrial antibody, *AMA*-*M2* anti-mitochondrial antibody-M2, *ASMA* anti-smooth muscle antibody, *LKM1* liver–kidney microsome type 1, *HLA* human leukocyte antigen

Normal ranges given in *parentheses*

**Fig. 2:**
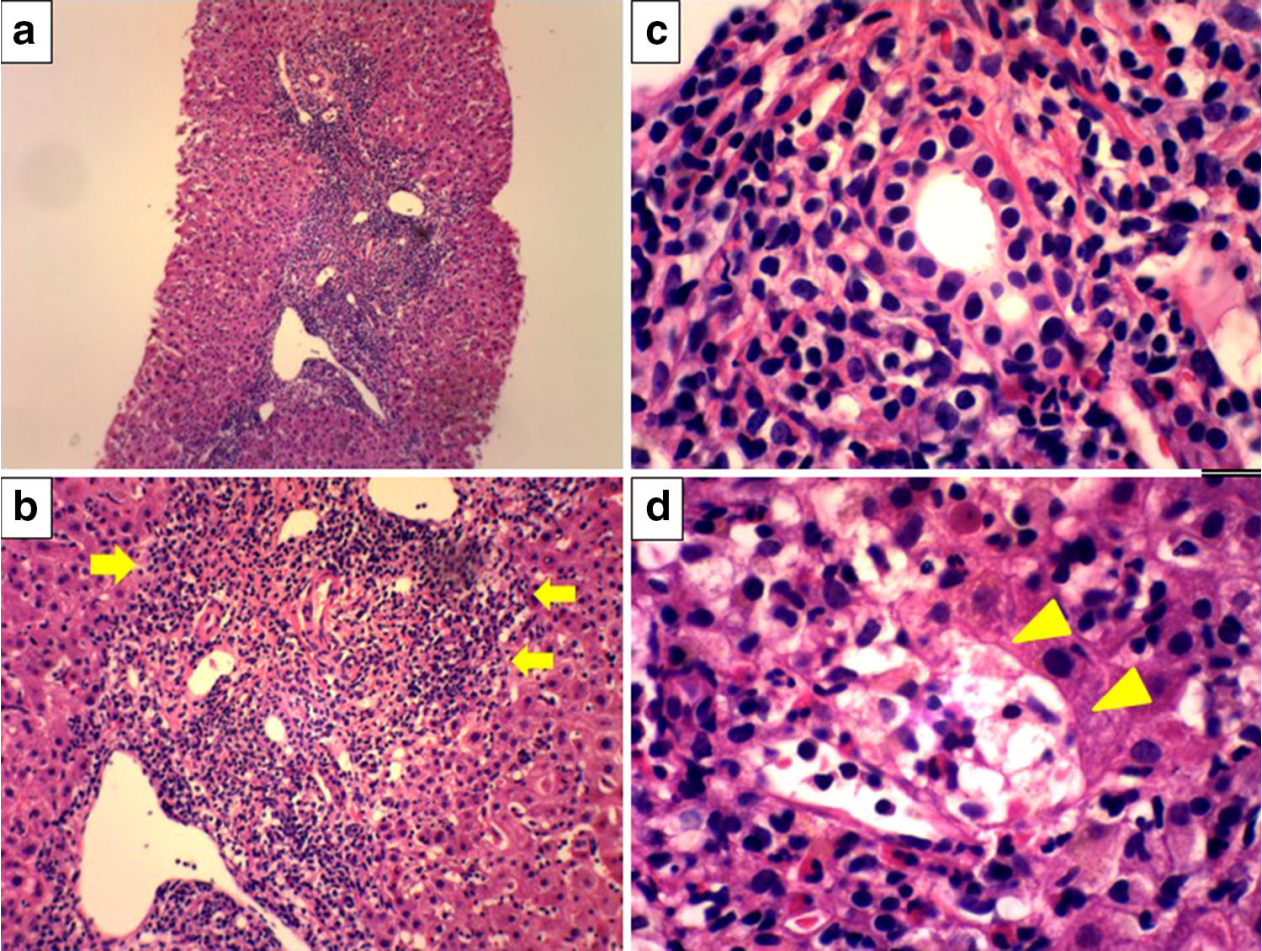
Liver histology for case 2 (daughter). **a** Liver biopsy showing severe chronic hepatitis with marked interface hepatitis (×2). **b** Magnified view of the liver biopsy showing dense lymphocytic/lymphoplasmacytic infiltrations in portal tracts and severe piecemeal necrosis (*arrow*, ×5). **c** Liver biopsy showing the absence of bile duct lesions, indicative of chronic nonsuppurative cholangitis and periductal granuloma (×20). **d** Severe intralobular necrosis and degeneration of hepatocytes (*arrowhead*, ×20)

**Fig. 3:**
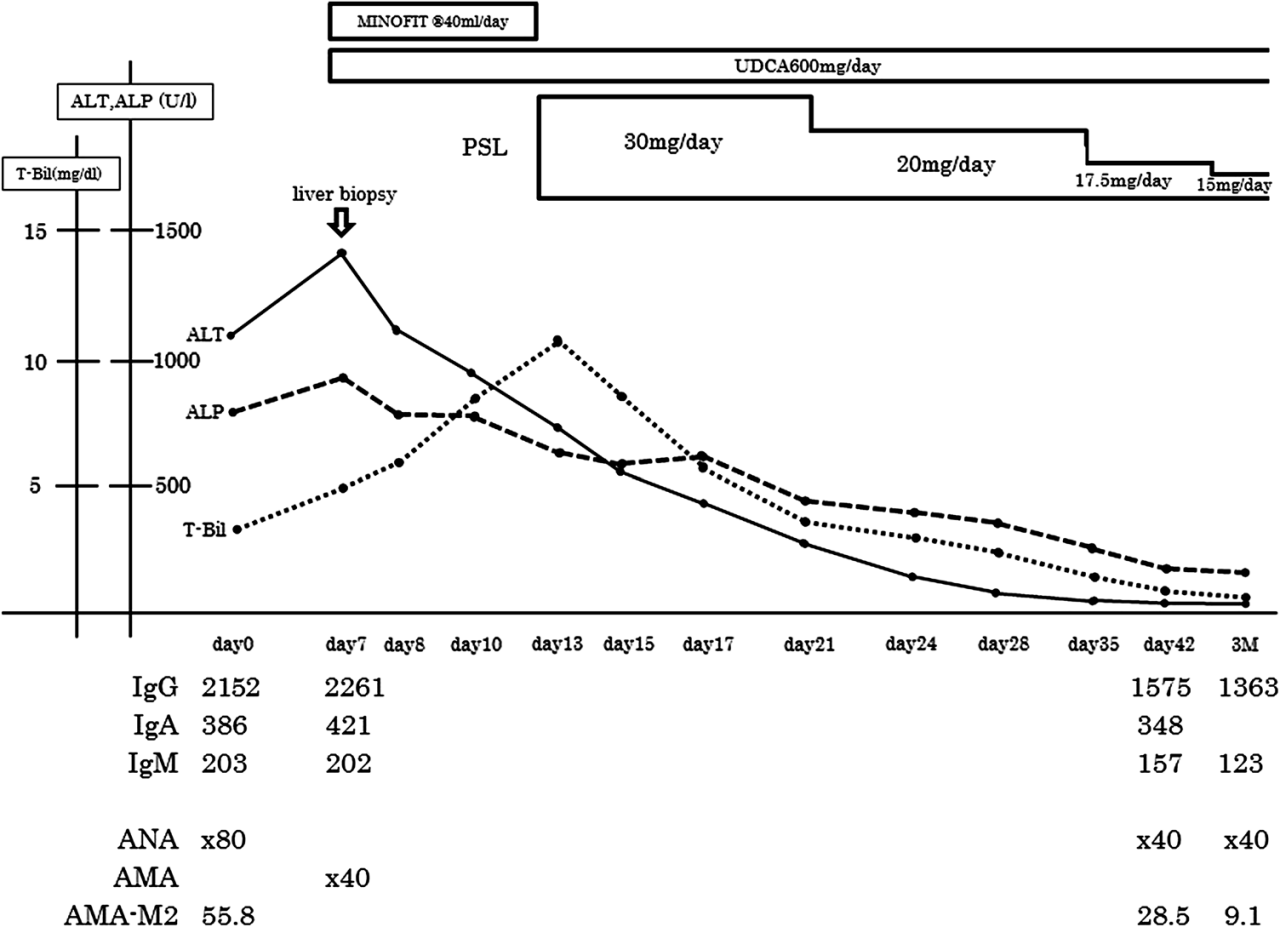
Clinical course of case 2 (daughter). Liver biopsy was performed on the day of admission. Following diagnosis with an autoimmune liver disease with overlapping features of primary biliary cholangitis and autoimmune hepatitis, the patient was treated with a combination of 600 mg/day ursodeoxycholic acid (*UDCA*) and 30 mg/day prednisolone (*PSL*) in accordance with the Japanese guidelines, resulting in an improvement of both clinical and biochemical findings within 1 month. Liver function has remained normal ever since, despite the subsequent tapering of PSL. Key: *ANA* antinuclear antibody, *AMA* anti-mitochondrial antibody, *AMA-M2* anti-mitochondrial antibody-M2

Case 2: a 49-year-old daughter was diagnosed with liver dysfunction for the first time during medical examinations. She had a variety of symptoms, including general fatigue and darkness of the urine for 2 weeks previously. She visited our hospital and was admitted because of visible jaundice and laboratory tests indicative of liver dysfunction: ALT 1425 U/L, AST 1203 U/L, ALP 900 U/L, γ-GTP 208 U/L, T-Bil 4.79 mg/dL, AMA titer 1:40, a positive AMA-M2 antibody, ANA 1:80 of the speckled type, and elevation of IgG to 2261 mg/dL (Table 1). Serological examinations for HAV, HBV, and HCV were all negative (Table 1). There was no history of symptoms suggestive of viral infection. The revised AIH score was 14 points, implying a probable AIH. However, the simplified IAIHG score was 7 points, implying a definite AIH [6, 7]. US and CT ruled out obstructive jaundice. The HLA-DRB1 haplotype was DRB1*04:05/DRB1*15:02, as observed with case 1. There was no history of medication use. Thus, we eventually diagnosed the patient, on the basis of both the Paris criteria and Japanese guidelines [4, 8], with an autoimmune liver disease with overlapping features of PBC and AIH. Pathological findings from liver biopsies revealed interface hepatitis with dense portal and periportal lymphocytic/lymphoplasmacytic infiltrations (Fig. 2). We immediately treated the patient with a combination of 600 mg/day UDCA and 30 mg/day PSL in accordance with the Japanese guidelines, which resulted in improvements of both clinical and biochemical findings within 1 month. Since then, liver function has remained normal, the current BMD is 0.508 g/cm^2^ greater than the normal range (0.416–0.488 g/cm^2^) and PSL has been slowly reduced to 7.5 mg (Fig. 3).

Familial studies (Table 1): the mother (case 1) has two children: the first daughter (case 2) and a second daughter. We tested liver function, autoantibodies, and HLA haplotype in all family members and subsequently compared their clinical backgrounds. The second daughter was healthy and had no history of liver dysfunction. Laboratory findings for the second daughter revealed normal liver function, a negative ANA, and a positive AMA-M2 antibody. The first daughter (case 2) had exactly the same HLA haplotype as the mother (case 1), whereas the second daughter’s HLA haplotype was DRB1*04:05/DRB1*01:01. There was no difference between any of the family members with respect to medication use, smoking status, alcohol consumption, or obstetric history. The period of time living with the mother for the first daughter (case 2) was same as that for the second daughter. The mother (case 1) was a housewife, which differed from both daughters’ occupations as nurses. There were no histories of symptoms suggestive of viral infection.

## Discussion

We encountered two patients exhibiting overlapping clinical features of PBC and AIH in a family with a similar genetic background in terms of HLA-DRB1 haplotype. Autoimmune liver disease exhibiting features of both PBC and AIH may be present at diagnosis or develop during follow-up. At diagnosis, our cases already fell into two diagnostic criteria: the Paris criteria and Japanese guidelines [4, 8]. In Japan, most patients with overlapping features of PBC and AIH have been reported to evolve into either PBC or AIH [5]. The development of AIH following liver transplantation for PBC has also been reported [10]. We suggest that our present cases provide further evidence of a link between these two diseases. Thus, autoimmune liver disease with overlapping features of PBC and AIH, the so-called “PBC–AIH overlap syndrome,” should no longer be considered as distinct diagnostic entities. However, PBC–AIH overlap syndrome has been reported to progress rapidly to cirrhosis and liver failure [11]. In our present cases, the revised AIH scoring system implied a probable AIH. On the other hand, based on the simplified IAIHG scoring system, a probable AIH was implied for case 1, and a definite AIH for case 2. The simplified IAIHG scoring system is thought to be more effective in diagnostic specificity than the revised AIH scoring system [4]. The relevant scoring system should therefore be selected depending on the situation and when considering the possibility of rapid progression when features of PBC and AIH overlap. In the Japanese guidelines, it is recommended that physicians treat PBC–AIH overlap syndrome with PSL in combination with UDCA when PBC patients have also been described to develop a probable or definite AIH based on the simplified IAIHG scoring system [4, 6, 12]. In addition, our cases were diagnosed as definite AIH using the revised AIH score, which has previously been considered to exclude a diagnosis of probable AIH for other liver disorders [7]. Although PBC–AIH overlap syndrome may not be considered as a distinct diagnostic entity, the therapeutic strategy indicated by the Japanese guidelines is considered to be a realistic and useful clinical policy to treat patients with overlapping features of PBC and AIH in a swift and effective manner, even if they occur within the same family, as observed in our current cases. Although our cases responded to PSL therapy, it is necessary to be careful when patients exhibit a high ALP level, negative for ASMA and positive for gp210. This is because these indications represent the possible risk that patients will be nonresponsive to PSL therapy, as reported previously [13]. Furthermore, the long-term use of PSL can cause adverse effects, including hyperglycemia, osteoporosis, cataracts, weight gain, and an increased risk of opportunistic infections. When treating patients presenting with overlapping features of PBC and AIH, physicians should be wary about such complications during follow-up. Fortunately, no adverse effects arose in either of our two patients as a result of the long-term use of PSL, although we will have to be careful with regard to the possibility of such complications arising over time. The current BMD levels in our two cases have remained higher than the normal range without alendronate treatment. However, the administration of alendronate may be necessary while monitoring BMD, as a previous study reported that alendronate led to the improvement of BMD in patients with PBC-related bone loss [14].

It has also been suggested that genetic susceptibility is an important factor in influencing the course of autoimmune liver disease and that HLA type is the most susceptible genetic factor responsible [1, 3]. Although PBC–AIH overlap syndrome in white individuals has been reported to be associated with positive tests for HLA-B8, DR3, or DR4, which are characterized as HLA haplotypes for AIH [2], both of our familial occurrence cases shared the same HLA haplotype, DRB1*04:05/DRB1*15:02. The second daughter’s HLA haplotype, however, was DRB1*04:05/DRB1*01:01. It is interesting to note that DRB1*04:05 was the same within all family members, whereas the DRB1*01:01 haplotype of the second daughter differed from the DRB1*15:02 haplotype exhibited by both case 1 and case 2. While it is well known that the frequency of the HLA-DRB1*04:05 haplotype is higher in Japanese AIH patients, it is difficult to accept that the HLA-DRB1*04:05 haplotype is associated with the susceptibility to develop overlapping features of PBC and AIH within the same family. This is because the second daughter, with a HLA-DRB1*04:05 haplotype, has not developed overlapping features of PBC and AIH in spite of a positive test for the AMA-M2 antibody. Furthermore, overlapping features of PBC and AIH within the same family are very rare in proportion to the high frequency of HLA-DRB1*04:05 in Japanese AIH patients. On the other hand, the association of DRB1*15:02, which differed from the second daughter’s HLA haplotype, with autoimmune liver disease, including the overlapping features of PBC and AIH within the same family, remains unknown, although DR15 has been reported to be associated with other autoimmune diseases such as systemic lupus erythematosus (SLE) [4, 15]. Earlier works have shown that SLE is associated with both AIH and PBC [16, 17]. Considering these conditions, it follows that the HLA-DRB1*15:02 haplotype may affect the susceptibility to develop overlapping features of PBC and AIH within the same family. None of our present family members had taken any medications to prevent such problems. Moreover, there are no means of predicting the onset of PBC and AIH overlap with respect to alcohol intake, smoking status, occupation, obstetric history, or viral serology. Although environmental conditions, the use of medications, and a variety of infectious factors were not considered to have influenced the onset of disease in either of our two patients, the possibility of their influences cannot be completely ruled out. As observed in our two patients, the exact mechanism underlying the onset of PBC and AIH overlap within the same family remains unclear. Therefore, it is very important to monitor the healthy second daughter closely as she was positive for the AMA-M2 antibody, as this might yield further knowledge with regard to what factors influence the onset of PBC and AIH overlap within the same family.

In case 2, pathological findings were compatible with AIH, in that there were no pathological findings of PBC, such as chronic nonsuppurative destructive cholangitis. Therefore, it was considered that AIH might represent a predominant pathophysiological factor in patients with overlapping features of PBC and AIH occurring within the same family, as observed in our cases. PBC patients with a genetic susceptibility to AIH may occasionally develop a mixed clinical picture of PBC and AIH. Existing reports also highlight that a genetic predisposition exists for both PBC and AIH, which share a common pathogenic mechanism [16].

The present cases suggest that a genetic predisposition is responsible for autoimmune liver disease with overlapping clinical features of PBC and AIH within the same family. It has also been reported that AIH develops among PBC patients after 6 months to 13 years [11]. As observed in our familial cases, the speed of sequential presentation of overlapping PBC and AIH features is very rapid. Consequently, autoimmune liver disease with clinical features of both PBC and AIH occurring within the same family should be treated very carefully by a combination of PSL and UDCA immediately, especially considering the possibility of rapid progression to liver cirrhosis and failure.
